# Supplementary material for: A Critical View of the Application of the APEX Software (Aqueous Photochemistry of Environmentally-Occurring Xenobiotics) to Predict Photoreaction Kinetics in Surface Freshwaters
Source: Molecules. 2019 Dec 18;25(1):9. doi: 10.3390/molecules25010009 (PMC7017383; doi:10.3390/molecules25010009)
Supplement: Supplementary file 1 [file molecules-25-00009-s001.zip › 3.2.4_gcc-4.4.0.zip!/3.2.4_gcc-4.4.0/3.2.4_gcc-4.4.0/share/octave/packages/symband-1.0.10/doc/SymBandDoc.pdf]

Symmetric Banded Matrices

Version 0.1 December, 2001
Andreas Stahel

June 7, 2009

Contents

1 Basic description **1**

2 Description of the commands **2**

2.1 `SBSolve` 2

2.2 `SBFactor` and `SBBacksub` 3

2.3 `SBEig` 4

2.4 `SBProd` 5

2.5 `BandToFull`, `FullToBand` and `BandToSparse` 6

1 Basic description

Many matrices used to solve PDE (using FEM) are symmetric. It the nodes are numbered properly then the matrix will show a band structure, i.e. all nonzero elements are located close to the main diagonal. The algorithm of Cholesky or the LDL^T factorization can take advantage of this structure, see [1]. For a symmetric matrix A of size $n \times n$ with semi-bandwidth b the approximate computational cost to solve one system of equations is given by

$$\text{Gauss} \approx \frac{1}{3}\,n^3 \;\; and \;\; \text{Band Cholesky} \approx \frac{1}{2}\,n\,b^2$$

Obviously for $b \ll n$ is is advantageous to use a banded solver. A more detailed analysis and an implementation is given in [2].

To take advantage of the symmetry and the band structure the matrices will be stored in a modified format, as illustrated below.

$$\left|\begin{array}{ccccc} 10 & 2 & 3 & 0 & 0 \\ 2 & 20 & 4 & 5 & 0 \\ 3 & 4 & 30 & 6 & 7 \\ 0 & 5 & 6 & 40 & 8 \\ 0 & 0 & 7 & 8 & 50 \end{array}\right| \longrightarrow \left|\begin{array}{ccc} 10 & 2 & 3 \\ 20 & 4 & 5 \\ 30 & 6 & 7 \\ 40 & 8 & 0 \\ 50 & 0 & 0 \end{array}\right|$$

A banded version of the LDL^T factorization in [1] can be implemented. If the matrix A is strictly positive definite, then the algorithm is known to be stable. If A is not positive definite, then problems might occur, since no pivoting is done. The matrix A is positive definite if and only if the diagonal matrix D is positive.

For a given matrix some of its smallest eigenvalues can be computed with an algorithm based on inverse power iteration. Precise information on the numerical errors is provided. The code is capable

Operations for symmetric, banded matrices	
`SBSolve()`	solve a system of linear equations
`SBFactor()`	find the $R^T DR$ factorization
`SBBacksub()`	use back-substitution to solve system of equations
`SBEig()`	find a few of the smallest eigenvalues and eigenvectors
`SBProd()`	multiply symmetric banded matrix with full matrix
`FullToBand()`	convert a symmetric matrix to a banded matrix
`BandToFull()`	convert a banded matrix to a symmetric matrix
`BandToSparse()`	convert a banded matrix to a sparse matrix

Table 1: List of commands

of finding eigenvalues of medium size matrices, where the standard command `eig()` is either very slow of will fail.

2 Description of the commands

2.1 `SBSolve`

The basic factorization algorithm is implemented in `SBSolve`. The function can return the solution of the system of linear equations, or the solution and the factorization of the original matrix. Multiple sets of equations can be solved.

```
[...] = SBSolve(...)
  solve a system of linear equations with a symmetric banded matrix

  X=SBSolve(A,B)
  [R,X]=SBSolve(A,B)

   solves A X = B

   A is mxt where t-1 is number of non-zero super-diagonals
   B is mxn
   X is mxn
   R is mxt

  if A would be ! 11000 ! then A= ! 11 !
                ! 14300 !         ! 43 !
                ! 03520 !         ! 52 !
                ! 00285 !         ! 85 !
                ! 00059 !         ! 90 !

  B is a full matrix

  The code is based on a LDL' decomposition (use L=R'), without pivoting.
  If A is positive definite, then it reduces to the Cholesky algorithm.
```

```
  R is an upper right band matrix
  The first column of R contains the entries of a diagonal matrix D.
  If the first column of R is filled by 1's, then we have R'*D*R = A
```

To determine the inverse matrix `A`$^{-1}$ one can use the command `invA = SBSolve(A,eye(n));`. Be aware that calculating the inverse matrix is rarely a wise thing to do. Most often the inverse of a banded matrix will loose the band structure.

If the matrix **A** is strictly positive definite, then the algorithm is stable and one can expect the solution to be as accurate as the conditions number of **A** permits. If **A** is semidefinite, then large errors might occur, since **not pivoting** is implemented in the code. The matrix is positive definite iff all eigenvalues are positive, this can be verified by inspection the sign of the numbers in the first column of **R**. The matrix is positive definite if the first column of the factorization matrix `R` (use `SBFactor()`) contains positive numbers only. A description of the algorithm can be found in [1] or [2].

2.2 SBFactor and SBBacksub

Instead of calling `X=SBSolve(A,B)` one can first call `R=SBFactor(A)` to determine the factorization $A = R^T DR$ and then `B=SBBacksub(R,X)` to solve the system(s) $A \cdot X = B$. Since most of the computational effort is in the factorization, this can be useful if many system of linear equations have to be solved sequentially. If multiple system are to be solved simultaneously it is preferable to use `SBSolve(A,B)` with a matrix `B` .

```
[...] = SBFactor(...)
  find the R'DR factorization of a symmetric banded matrix

  R=SBFactor(A)

   A is mxt where t-1 is number of non-zero super diagonals
   R is mxt

  if A would be ! 11000 ! then A= ! 11 !
                ! 14300 !         ! 43 !
                ! 03520 !         ! 52 !
                ! 00285 !         ! 85 !
                ! 00059 !         ! 90 !


  The code is based on a LDL' decomposition (use L=R'), without pivoting.
  If A is positive definite, then it reduces to the Cholesky algorithm.

  R is an upper right band matrix
  The first column of R contains the entries of a diagonal matrix D.
  If the first column of R is filled by 1's, then we have R'*D*R = A

[...] = SBBacksub(...)
  using backsubstitution  to return the solution of a system of linear equations

  X=SBBacksub(R,B)

   B is mxn
```

```
X is mxn
R is mxt

R is produced by a call of [X,R] = SBSolve(A,B) or R = SBFactor(A)
It is an upper right band matrix
The first column of R contains the entries of a diagonal matrix D.
If the first column of R is filled by 1's, then we have R'*D*R = A
```

If there is interest in the classical Cholesky decomposition of the matrix A (i.e. $A = R' \cdot R$) then R can be computed by

```
rBand=SBFactor(A);
d=sqrt(rBand(:,1));
rBand(:,1)=ones(n,1);
r=triu(diag(d)*rBand)
```

The number of positive/negative numbers in the first column of **R** equals the number of positive/negative eigenvalues of **A**.

2.3 SBEig

For given symmetric matrices **A** and **B** the standard (resp. generalized) eigenvalue problem will be solved, i.e.

$$\mathbf{A}\,\vec{v} = \lambda\,\vec{v}\ \textit{resp.}\ \mathbf{A}\,\vec{v} = \lambda\,\mathbf{B}\,\vec{v}$$

Using inverse power iteration a given number of the smallest (absolute value) eigenvalues if a symmetric matrix **A** are computed. If needed the eigenvectors are also generated. A set of initial vectors **V** have to be given. If those are already close to the eigenvectors, then the algorithm will converge rather quickly. For a precise description and analysis consult [1].

```
[...] = SBEig(...)
  find a few eigenvalues of the symmetric, banded matrix
  inverse power iteration is used for the standard and generalized
  eigenvalue problem

  [Lambda,{Ev,err}] = SBEig(A,V,tol)     solve A*Ev = Ev*diag(Lambda)
                    standard eigenvalue problem

  [Lambda,{Ev,err}] = SBEig(A,B,V,tol)   solve A*Ev = B*Ev*diag(Lambda)
                    generalized eigenvalue problem

   A   is mxt, where t-1 is number of non-zero superdiagonals
   B   is mxs, where s-1 is number of non-zero superdiagonals
   V   is mxn, where n is the number of eigenvalues desired
       contains the initial eigenvectors for the iteration
   tol is the relative error, used as the stopping criterion

   X   is a column vector with the eigenvalues
   Ev  is a matrix whose columns represent normalized eigenvectors
   err is a vector with the a posteriori error estimates for the eigenvalues
```

The algorithm is based on inverse power iteration with n independent vectors. The iteration will proceed until the relative change of all eigenvalues is smaller than the given value of `tol`. This does not guarantee that the relative error is smaller than `tol`. The initial guesses $\mathbf{V}$ for the eigenvectors have to be linearly independent. The closer the initial guess is to the actual eigenvector,the faster the algorithm will converge. The algorithm returns n eigenvalues closest to 0 .

For the standard eigenvalue problem $\mathbf{A}\,\vec{v}_i = \lambda_i\,\vec{v}_i$ the eigenvectors $\vec{v}_i$ will be orthonormal with respect to the standard scalar product, i.e, $\langle \vec{v}_i\,,\,\vec{v}_j\rangle = \delta_{i,j}$. For the generalized eigenvalue problem $\mathbf{A}\,\vec{v}_i = \lambda_i\,\mathbf{B}\,\vec{v}_i$ this translates to $\langle \vec{v}_i\,,\,\mathbf{B}\,\vec{v}_j\rangle = \delta_{i,j}$. The symmetric matrix $\mathbf{B}$ should be positive definite. The columns of `Ev` can be used to restart the algorithm if higher accuracy is required.

The algorithm will return reliable estimates for the errors in the eigenvalues. The à posteriori error estimate is based on the residual $\vec{r} = \mathbf{A}\,\vec{v} - \lambda\,\vec{v}$ and

$$\min_{\lambda_i \in \sigma(\mathbf{A})} |\lambda - \lambda_i| \leq \langle \vec{r}\,,\,\vec{r}\rangle = \|\vec{r}\|$$

where we use the normalization $\langle \vec{v}\,,\,\vec{v}\rangle = 1$. If one of the eigenvalues has to be computed with high accuracy, the approximate value λ may be subtracted from the diagonal of the matrix. Then the eigenvalue closest to zero of the modified matrix $\mathbf{A} - \lambda\mathbb{I}$ can be computed, using the already computed eigenvector. If the eigenvalue is isolated the algorithm will converge very quickly. This algorithm is similar to the Rayleigh quotient iteration. A good description is given in [1].

If the eigenvalue closest to λ is denoted by λ_i we have the improved estimate

$$|\lambda - \lambda_i| \leq \frac{\|\vec{r}\|^2}{\text{gap}} \; \textit{where} \; \text{gap} = \min\{|\lambda - \lambda_j| \;:\; \lambda_j \in \sigma(\mathbf{A}), j \neq i\}$$

It is very easy to implement this test in *Octave*. If the estimate is based on approximate values of the eigenvalues, then the result is not as reliable as the previous one. Since the value of `gap` will carry an approximation error. The situation is particularly bad if some eigenvalues are clustered. A code sample is provided.

For the generalized eigenvalue problem we use the residual $\vec{r} = \mathbf{A}\,\vec{v} - \lambda\,\mathbf{B}\,\vec{v}$ and the estimate

$$\min_{\lambda_i \in \sigma(\mathbf{A})} |\lambda - \lambda_i| \leq \sqrt{\langle \vec{r}\,,\,\mathbf{B}^{-1}\vec{r}\rangle} \; \textit{and} \; |\lambda - \lambda_i| \leq \frac{\|\vec{r}\|^2}{\text{gap}}$$

where we use the normalization $\langle \vec{v}\,,\,\mathbf{B}\,\vec{v}\rangle = 1$. The precise algorithm and proof of the above estimate is given in [2].

2.4 SBProd

With this command a symmetric banded matrix can be multiplied with a full matrix.

```
[...] = SBProd(...)
  multiplies a symmetric banded matrix with a matrix

  X=SBProd(A,B)

   A is mxt where t-1 is number of non-zero super diagonals
   B is mxn
   X is mxn

  if A would be ! 11000 ! then A= ! 11 !
```

```
! 14300 !          ! 43 !
! 03520 !          ! 52 !
! 00285 !          ! 85 !
! 00059 !          ! 90 !

B is full matrix Ax=B
```

2.5 `BandToFull`, `FullToBand` and `BandToSparse`

With these commands conversion between full, symmetric matrices and banded symmetric matrices is possible. A conversion to a sparse format is also included.

References

[1] G. Golub and C. Van Loan, *Matrix computations*, John Hopkins University Press, third edition, 1996

[2] A. Stahel, *Calculus of Variations and Finite Elements*, Lecture notes used at HTA Biel, 2000
